# PSCP, a novel reactive sulfur donor, activates Keap1-Nrf2 signaling and attenuates mitochondrial dysfunction in diabetic retinopathy

**DOI:** 10.3389/fendo.2025.1690553

**Published:** 2025-11-17

**Authors:** Kexin Li, Ruiying Ji, Youbang Chen, Yiwen Wang, Yiyan Guo, Xiang Li, Hui Zhang, Liang Guo, Chun-tao Yang

**Affiliations:** 1 Guangzhou Municipal and Guangdong Provincial Key Laboratory of Protein Modification and Disease, School of Basic Medical Sciences, Guangzhou Medical University, Guangzhou, China; 2 School of Exercise and Health and Collaborative Innovation Center for Sports and Public Health, Shanghai University of Sport, Shanghai, China

**Keywords:** reactive sulfur species, diabetic retinopathy, mitochondrial dysfunction, Keap1-Nrf2 pathway, MGST1, IDH2

## Abstract

**Objective:**

Diabetic retinopathy (DR) is a leading cause of vision loss in diabetes, yet its underlying molecular drivers remain poorly defined. This study aimed to identify diabetic stress-responsive targets and explore the therapeutic potential of the reactive sulfur donor PSCP by integrating transcriptomic and functional analyses in diabetic mouse and cell models.

**Methods:**

Retinal transcriptomic datasets from type 1 and type 2 diabetic mice (GSE111465 and GSE55389) were analyzed for mitochondrial and antioxidant gene expression. *In vitro*, ARPE-19 retinal pigment epithelial cells were exposed to hydrogen peroxide (H_2_O_2_) or methylglyoxal (MGO) to induce oxidative and carbonyl stress. Mitochondrial function, gene expression, and antioxidant pathway activation were assessed in the presence or absence of PSCP or/and Nrf2 inhibitor ML385.

**Results:**

Transcriptomic analysis revealed consistent dysregulation of mitochondrial antioxidant enzymes IDH2 and MGST1 in diabetic retinas. Oxidative and carbonyl stress in ARPE-19 cells led to reactive oxygen species accumulation, loss of mitochondrial membrane potential, and reduced cell viability, accompanied by suppression of IDH2 and MGST1. PSCP treatment induced Keap1 modification and promoted Nrf2 nuclear translocation, restoring the expression of IDH2, MGST1, and mitochondrial dynamics regulators MFN2 and FIS1. PSCP also preserved mitochondrial membrane potential and improved cell survival. This protective effect was abrogated by ML385.

**Conclusions:**

Our findings identify IDH2 and MGST1 as stress-responsive mitochondrial targets in DR and demonstrate that PSCP activates the Keap1-Nrf2 pathway to preserve mitochondrial integrity under diabetic stress.

## Highlights

- Mitochondrial dysfunction is a converging feature in diabetic retinopathy.
- Aberrant expressions of MGST1 and IDH2 disrupt mitochondrial redox regulation.
- PSCP, a novel reactive sulfur donor, activates Keap1-Nrf2 signaling.
- PSCP-induced Nrf2 activation restores mitochondrial homeostasis and protects retinal cells from diabetic stress.

## 1 Introduction

Diabetic retinopathy (DR) is a common complication of diabetes mellitus (DM) and remains the leading cause of blindness among diabetic patients (1). One of the major challenges in treating DR is its gradual onset, as visual deficits are often overlooked in the early stages (2, 3). In many cases, retinal impairment remains undetected until formal fundus screening is performed, highlighting the urgent need to identify novel biomarkers and molecular signatures for early intervention. The multifactorial nature of DM-induced retinal damage makes it difficult to attribute the condition to a single pathogenic factor (4); therefore, it is imperative to develop effective and feasible therapeutic strategies.

To advance DR research and explore potential therapeutic interventions, appropriate diabetic models are essential. Currently, two primary animal models are widely used: the streptozotocin (STZ)-induced type 1 diabetes mellitus (T1DM) model and the *db/db* mouse model of type 2 diabetes mellitus (T2DM) (5, 6). These models exhibit distinct pathological mechanisms and disease progression patterns. The STZ-induced model mimics T1DM by selectively destroying pancreatic β-cells, leading to insulin deficiency and hyperglycemia (5). In contrast, the *db/db* mouse model, characterized by a mutation in leptin receptor gene, develops obesity-induced insulin resistance, resembling the natural progression of T2DM (6). Despite their widespread use, the extent to which these models accurately reflect the clinical course of diabetes and its complications, particularly DR, remains an area of debate. The differences in disease onset, metabolic profiles between these models may influence their applicability to DR research (7). Therefore, a critical evaluation of their advantages and limitations is essential for selecting the most appropriate model for studying the pathogenesis of DR and developing therapeutic approaches.

The pathophysiology of DR is closely associated with DM-triggered metabolic dysregulation, which promotes the excessive accumulation of reactive oxygen species (ROS), as well as reactive aldehydes like methylglyoxal (MGO) (8, 9). These highly reactive compounds facilitate the formation of advanced glycation end products (AGEs) through non-enzymatic glycation of proteins and nucleic acids, leading to structural and functional deterioration (10). Substantial evidence indicates that oxidative insults disrupt cellular homeostasis, and accelerate neurovascular degeneration, collectively driving DR progression (11–13). However, their precise mechanisms remain incompletely understood, and further investigations are still needed to advance the development of individualized therapies for DR.

Bioinformatics has revolutionized biomedical research by integrating computational analysis with high-throughput biological data, facilitating the discovery of key molecular drivers of disease (14). Through advanced algorithms and data visualization techniques, bioinformatics enables the identification of latent patterns in large-scale datasets, guiding both basic research and clinical decision-making. Gene set enrichment analysis (GSEA) is a powerful computational approach for uncovering signaling pathways and phenotypic alterations (15, 16). In this study, we leveraged publicly available transcriptomic datasets from the Gene Expression Omnibus (GEO) to systematically characterize gene expression patterns and functional pathways in the retinas of diabetic mouse models. Furthermore, we validated these pathways using cell-based and molecular biology experiments, providing a solid framework for elucidating the mechanisms of DR pathogenesis and identifying novel therapeutic targets.

## 2 Materials and methods

### 2.1 Expression data of diabetic retinal tissues

Gene expression microarray datasets were obtained from the GEO database. The GSE55389 microarray comprised eight retinal tissue samples: four from T2DM mice and four from control mice (https://www.ncbi.nlm.nih.gov/geo/query/acc.cgi?acc=GSE55389). The GSE111465 microarray contained 12 retinal tissue samples: six T1DM mice and six from control mice (https://www.ncbi.nlm.nih.gov/geo/query/acc.cgi?acc=GSE111465). The T2DM mouse model was established through the defective leptin receptor gene (*db*). C57BLKsJ mice with homozygous mutation (*db/db*) developed typical diabetic manifestations, while heterozygous mice (*db/m*+) exhibited normal phenotypes. Total RNA from retinal tissues was extracted using TRIzol reagent for reverse transcription, followed by hybridization with MoGene-1_0-st DNA arrays (Affymetrix, UK) (6). The T1DM mouse model was generated through intraperitoneal injection of STZ (160 mg/kg body weight; Sigma) into 8-week-old C57BL6/J mice. Control mice received an equivalent volume of normal saline. Mice were considered diabetic when fasting blood glucose exceeded 15 mM. After 6 weeks, total RNA from retinal tissues was extracted and reverse-transcribed, and the resulting cDNA was hybridized with MoGene-2_0-st microarray.

### 2.2 Quality control and clustering of RNA expression

Both microarray datasets were imported into the R-4.4.3 platform for analysis. Boxplots displaying extreme values, median, and quartiles were generated to visualize the overall gene expression distribution in each sample. Dimensionality reduction algorithms were applied to visually analyze data clustering patterns with the t-distributed Stochastic Neighbor Embedding (t-SNE) R package (17).

### 2.3 Differential expression and functional enrichment analysis

Differential gene expression analysis between diabetic and control groups was performed using the limma R package. Genes exhibiting differential expression at *P* < 0.05 were selected for downstream analysis. The distribution of these differentially expressed genes was visualized using volcano plots generated with the ggplot2 R package, allowing for simultaneous visualization of statistical significance and magnitude of change.

For enrichment analysis, GSEA was conducted using a gene list ranked by log2 fold change (FC) in descending order (15). The GSEA implementation utilized the clusterProfiler and fgsea R packages to evaluate coordinated expression changes. The analysis referenced the MSigDB mouse gene symbol collection (m2.all.v2024.1.Mm.symbols.gmt) to identify enriched pathways. Statistical significance was assessed after multiple testing correction using the Benjamini-Hochberg (BH) procedure. Pathway enrichment was quantified using normalized enrichment scores (NES) and P values. The GseaVis package was employed to generate enrichment plots for pathways of interest, illustrating the distribution of member genes within the ranked list and their contributions to the enrichment signal.

### 2.4 Cell culture

ARPE-19 cells, a spontaneously immortalized human retinal pigment epithelium cell line (18), were obtained from Meisen Chinese Tissue Culture Collections (Jinhua, China). Cells were cultured in DMEM-F12 medium supplemented with 10% fetal bovine serum (Tianhang Biotechnology Co., Ltd, Huzhou, China). Cultures were maintained at 37 °C in a humidified atmosphere containing 5% CO_2_ and 95% air. Cells were sub-cultured at 80-90% confluency using 0.25% trypsin-EDTA solution and passaged every 48 hours.

### 2.5 Cell viability assay

Cell viability was assessed using the Cell Counting Kit-8 (CCK-8) (Dojindo Lab., Kyushu, Japan). ARPE-19 cells were seeded in 96-well plates at a density of 1×10^4^ cells per well and treated accordingly when they reached approximately 70% confluence. Following the indicated treatments, 10 μL of CCK-8 solution diluted in 100 μL of serum-free DMEM-F12 medium was added to each well, and the plates were incubated at 37 °C for 2 h in the dark. Absorbance (A) at 450 nm was measured using an Infinite M200 PRO microplate reader (Tecan Group Ltd., Männedorf, Switzerland). Cell viability was calculated using the following equation: Cell viability (%) = (*A*_Treatment group_ – *A*_Blank_)/(*A*_Control group_ – *A*_Blank_) × 100.

### 2.6 Western blot analysis

The expression levels of p53, IDH2, MGST1, FIS1, and MFN2 in ARPE-19 cells were assessed using Western blot analysis. After the indicated treatments, cells were harvested and lysed on ice, using RIPA buffer (50 mM Tris-HCl pH 7.4, 150 mM NaCl, 1% NP-40, 0.5% sodium deoxycholate, 0.1% SDS) supplemented with protease and phosphatase inhibitor cocktails, and protein concentrations were determined with a BCA protein assay kit. Equal amounts of protein from each sample were separated via SDS-PAGE and subsequently transferred onto PVDF membranes. The membranes were then blocked with 5% fat-free milk in Tris-buffered saline containing 0.1% Tween-20 (TBS-T) for 1 h at room temperature. Following blocking, the membranes were incubated with primary antibodies under gentle agitation overnight at 4 °C. After extensive washing, horseradish peroxidase (HRP)-conjugated secondary antibodies were applied for 1 h at room temperature. Protein signals were visualized using an enhanced chemiluminescence (ECL) detection system, and band intensities were quantified using ImageJ software.

### 2.7 Oxidative stress and mitochondrial function assessment

Intracellular ROS levels were measured using a dichloro-dihydro-fluorescein diacetate (DCFH-DA) assay kit (Dojindo Lab., Kyushu, Japan). ARPE-19 cells were seeded in 48-well plates and allowed to grow until reaching approximately 70% confluence. Cells were then subjected to the indicated treatments. Then, 200 μL of DCFH-DA working solution was added to each well, and the cells were incubated at 37 °C for 30 min in the dark. The fluorescence intensity of DCF, representing ROS accumulation, was visualized using an inverted fluorescence microscope (Carl Zeiss AG, Oberkochen, Germany).

The mitochondrial membrane potential (MMP) was assessed using JC-1 staining (Dojindo, Kumamoto, Japan). ARPE-19 cells were treated with MGO in the absence or presence of PSCP, followed by incubation with JC-1 working solution at 37 °C for 40 min in the dark. After washing with buffer, fluorescence images were captured under a confocal microscope (Zeiss, Germany), where red and green fluorescence represented JC-1 aggregates and monomers, respectively, reflecting changes in MMP.

The levels of oxidized glutathione (GSSG) in ARPE-19 cells were measured using a GSH detection kit (Servicebio, Wuhan, China). ARPE-19 cells were cultured in six-well plates at 37 °C with 5% CO_2_ until reaching approximately 50% confluence. After washing with PBS, the cells were collected by centrifugation (200 g, 5 min). The cell pellets were resuspended in protein removal reagent and lysed via sonication or ice incubation (15 min), followed by centrifugation (10,000 g, 15 min, 4 °C). Before GSSG quantification, GSH was first eliminated by adding 5 μL of GSH scavenger to 50 μL sample. Subsequently, GSSG was converted to GSH using NADPH and glutathione reductase. Following conversion, 20 μL of the treated sample was combined with 160 μL of glutathione detection working solution and incubated at room temperature for 5 min. After adding 20 μL of substrate solution, the reaction was incubated at 25 °C for 20 min. Absorbance was measured at 412 nm using the above microplate reader. GSSG content was calculated based on the GSH measured post-conversion.

### 2.8 Apoptosis detection

Apoptotic cells were detected using Hoechst 33342 staining (Beyotime Biotechnology, Shanghai, China). ARPE-19 cells were treated with 400 μM MGO for 48 h in the presence or absence of the p53 inhibitor PFT-α (20 μM). After treatment, cells were washed twice with PBS and incubated with Hoechst 33342 (10 μg/mL) for 10 min at 37 °C in the dark. Cells were then washed with PBS and immediately observed under a fluorescence microscope. Apoptotic cells were identified by characteristic nuclear morphological changes, including chromatin condensation, nuclear fragmentation, and formation of apoptotic bodies. Images were captured and the percentage of apoptotic cells was calculated using ImageJ software.

### 2.9 Nuclear translocation of Nrf2

ARPE-19 cells were cultured on coverslips. At approximately 70% confluence, the cells were treated accordingly. Then, they were fixed with cold methanol at -20 °C for 5 min and blocked with 5% goat serum at room temperature for 40 min. Subsequently, the cells were incubated with a primary antibody against Nrf2 (1:500; No. Ab62352, Abcam, USA) at 4 °C overnight. After washing, an Alexa Fluor^®^ 594-conjugated goat anti-rabbit IgG was applied. Following PBS washes, the samples were mounted using an antifade mounting medium containing 4’,6-diamidino-2-phenylindole (DAPI). The signals were visualized using the above fluorescence microscope.

### 2.10 Statistical analysis

Experimental data are presented as mean ± standard deviation (SD). Statistical significance between groups was evaluated by one-way analysis of variance (ANOVA) followed by the Student-Newman-Keuls test using GraphPad Prism 9.0 software (San Diego, USA). A probability value < 0.05 was considered statistically significant. The R programming language was utilized for advanced statistical analyses, including clustering, function enrichment, feature screening and data visualization.

## 3 Results

### 3.1 Quality control and dimensionality reduction clustering analysis of gene expression in retinal tissues of T1DM and T2DM mice

**Figure 1:**
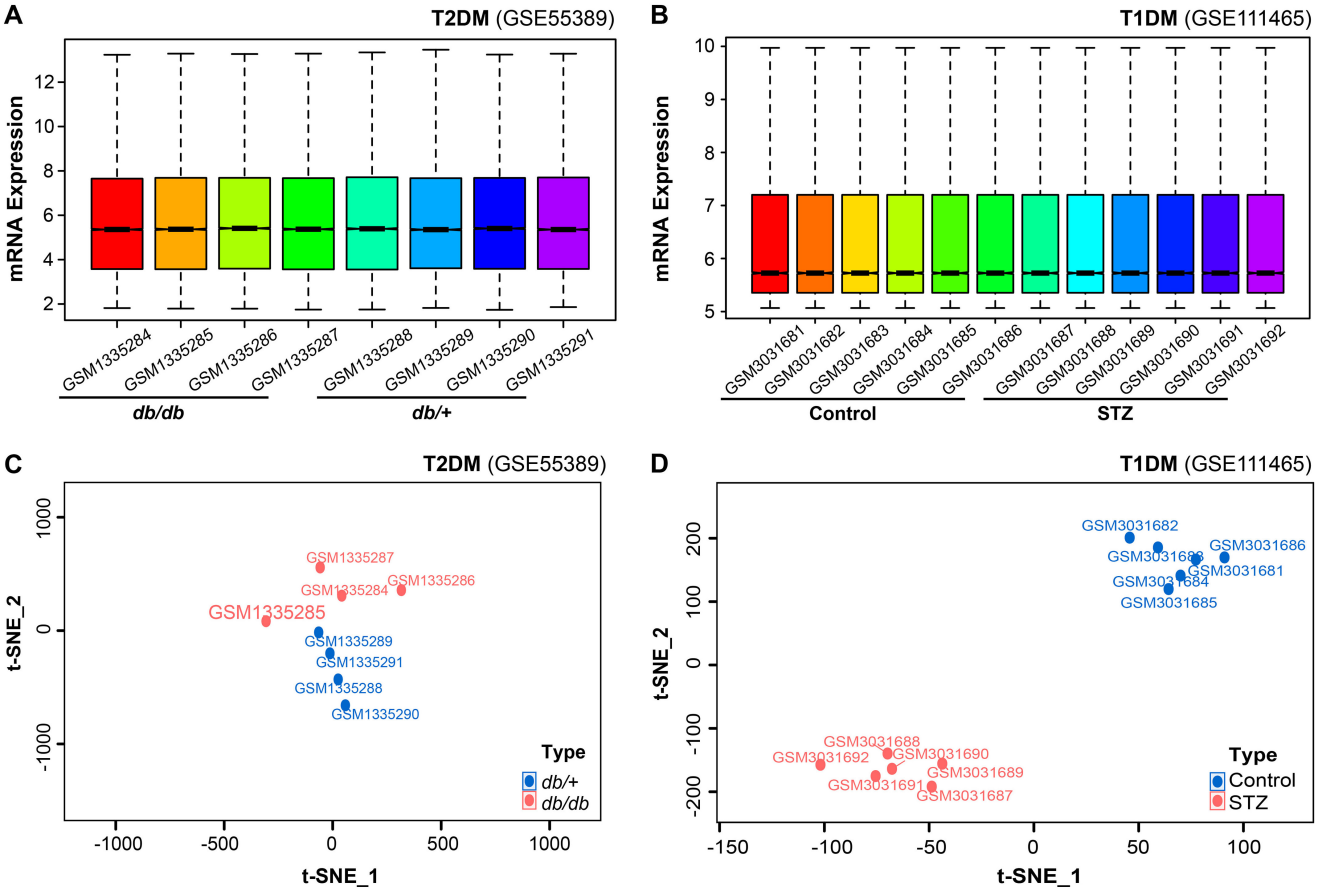
Comprehensive bioinformatics analysis of retinal mRNA expression in diabetic mouse models. **(A, B)** Quality control assessment and expression profiling of total mRNA in the microarray datasets GSE55389 [T2DM retinal tissues, **(A)**] and GSE111465 [T1DM retinal tissues, **(B)**]. **(C, D)** t-SNE-based dimensionality reduction and clustering analysis of mRNA expression data from GSE55389 [T2DM, **(C)**] and GSE111465 [T1DM, **(D)**].

Analysis of the GSE55389 and GSE111465 microarray datasets revealed distinct transcriptomic patterns in T2DM and T1DM retinal tissues. Following normalization and log2 transformation, boxplot analyses revealed no significant differences in total mRNA expression levels between diabetic and control tissues (**Figures 1A, B**). Dimensionality reduction and clustering analysis were performed on the mRNA expression data from both microarrays using the t-SNE algorithm. As shown in **Figure 1C**, the two groups from the GSE55389 dataset (T2DM and Control) exhibited partial overlap in the t-SNE plot. In contrast, analysis of the GSE111465 dataset demonstrated clear clustering separation between the T1DM and Control samples (**Figure 1D**), indicating distinct gene expression patterns. Based on these analyses, both datasets were deemed suitable for subsequent differential expression analyses.

### 3.2 Identification of differentially expressed genes and functional enrichment analysis in diabetic retinal tissues

**Figure 2:**
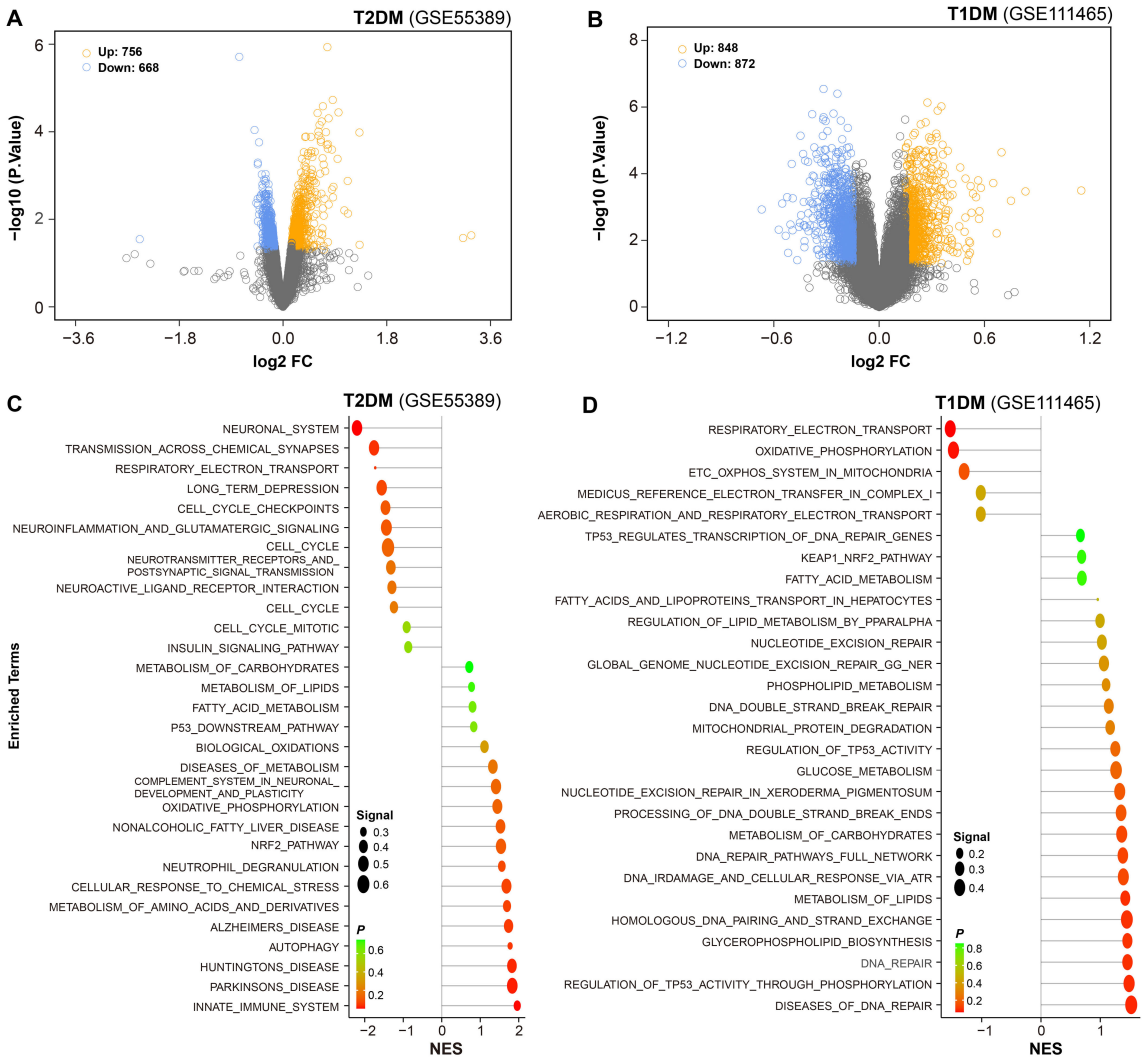
Differential gene expression and pathway enrichment in diabetic retinal tissues. **(A, B)** Volcano plots were employed to show differential gene expression in retinal tissues of T2DM and T1DM mice. Yellow and blue dots represented significantly upregulated and downregulated genes, respectively. **(C, D)** Functional enrichment analysis of the differentially expressed genes was performed, with reference to the Molecular Signatures Database (MSigDB), to identify enriched biological pathways.

Differentially expressed genes were identified based on a significance threshold (*P* = 0.05). In the microarray dataset GSE55389, a total of 756 genes were found to be upregulated, while 668 genes were downregulated in the retinal tissues of T2DM mice (**Figure 2A**). Under the same criteria, 848 genes were upregulated and 872 genes were downregulated in the retinal tissues of T1DM mice (**Figure 2B**).

To further elucidate the biological significance of these DEGs, we conducted functional enrichment analysis. The results revealed that in the retinal tissues of T2DM mice, the upregulated genes were predominantly associated with pathways related to neurodegenerative changes, innate immune response, and metabolic dysregulation, such as biological oxidation and oxidative phosphorylation. The downregulated genes were involved in neurotransmission (**Figure 2C**).

In contrast, the retinal tissues of T1DM mice exhibited distinct patterns of pathway enrichment. The upregulated genes were primarily associated with DNA repair, lipid metabolism, and stress responses, such as p53-regulated transcription, fatty acid metabolism, and KEAP1-Nrf2 pathway. Importantly, beyond these distinct enrichments, several common pathways were shared between T1DM and T2DM mice, including NRF2 pathway, metabolism of lipid, and fatty acid metabolism, highlighting overlapping pathogenic mechanisms across different diabetic models. The downregulated genes were involved in mitochondrial function, like energy metabolism, respiratory electron transport and oxidative phosphorylation (**Figure 2D**).

These findings suggest that both types of diabetes lead to significant alterations in multiple key biological processes within the retinal tissues, but with distinct patterns of pathway dysregulation. The identification of these pathways provides valuable insights into the pathogenesis of diabetic retinopathy and offers potential targets for therapeutic intervention.

### 3.3 Analysis of diabetes-induced neuronal pathway alterations in mouse retinal tissues

**Figure 3:**
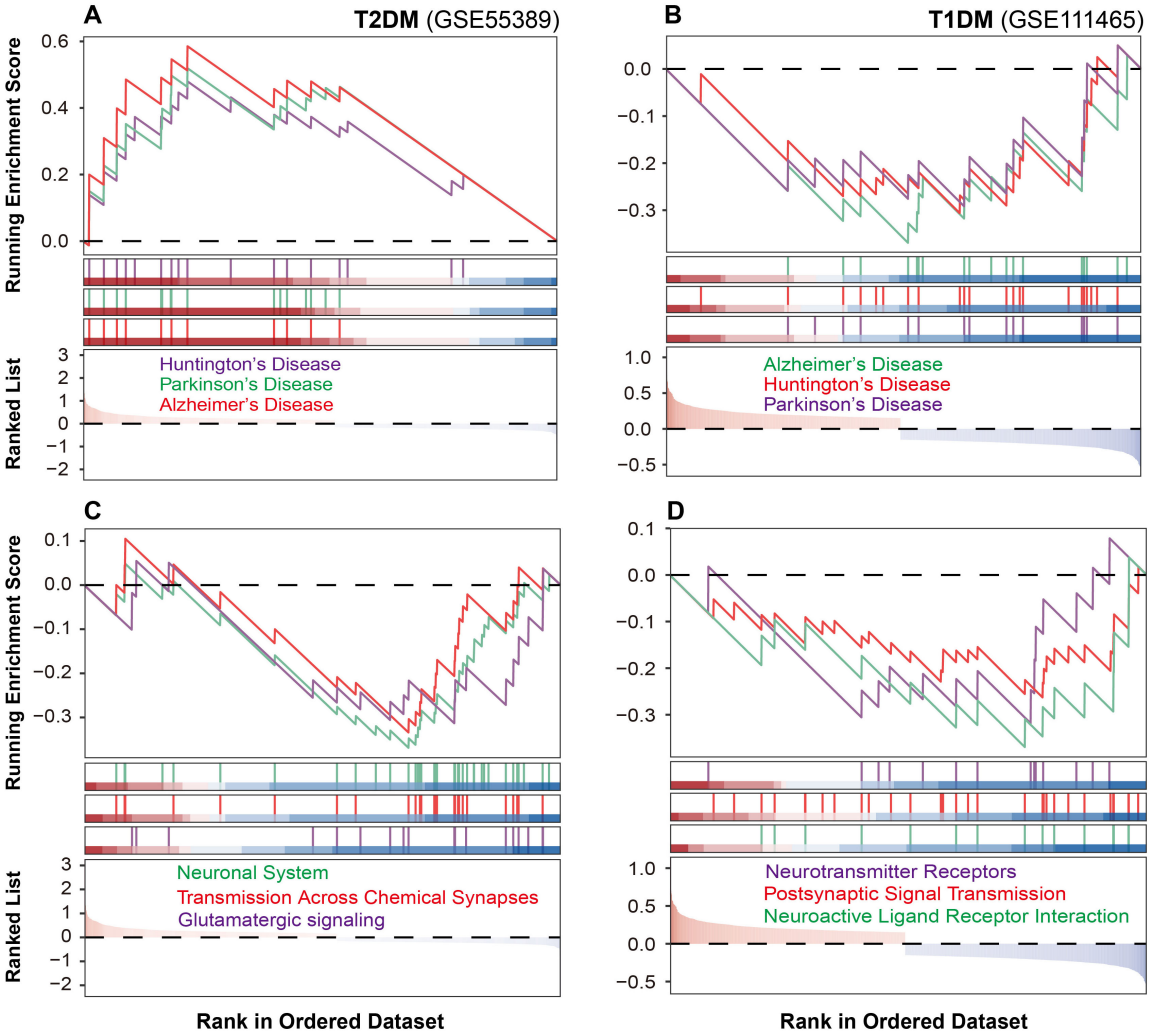
Neuronal pathway alterations in diabetic mouse retinal tissues. **(A, B)** Neurodegenerative pathway alterations in T2DM and T1DM mouse retinal tissues. **(C, D)** Neuronal communication pathway changes in T2DM and T1DM.

Pathway enrichment analysis revealed distinct molecular alterations in retinal tissues between T1DM and T2DM. To gain deeper insights into these distinctions, we performed a functional classification-based enrichment analysis. In T2DM, pathways associated with neurodegenerative disorders, including Alzheimer’s, Parkinson’s, and Huntington’s diseases, were significantly upregulated (**Figure 3A**). Conversely, these pathways were downregulated in T1DM (**Figure 3B**).

As the neurodegeneration often converges on the disruption of synaptic transmission and signal integration, we next examined the integrity of neuronal communication networks. T2DM was characterized by marked downregulation of glutamatergic signaling, chemical synaptic transmission, and neurotransmitter transport pathways (**Figure 3C**). T1DM similarly demonstrated disrupted neural function, but through significant suppression of neuroactive ligand-receptor interactions, postsynaptic signaling, and neurotransmitter receptor activity (**Figure 3D**). These findings suggest that although both T1DM and T2DM compromise neuronal communication, they do so via distinct molecular pathways.

Given that many of these transcriptomic changes are due to cellular stress responses, especially oxidative stress, we selected retinal pigment epithelium (RPE) cells as a model to further investigate the underlying mechanisms and potential protective strategies. RPE cells were chosen because: (1) they provide essential metabolic support and nutritional supply to photoreceptors, making them a key cellular target in retinal disease research; (2) RPE dysfunction is a critical early event in diabetic retinopathy pathogenesis; and (3) they are highly vulnerable to oxidative and carbonyl stress, which aligns with our identified pathways of glutathione metabolism and mitochondrial function. While photoreceptor cells are also important candidates for DR research, RPE cells are more widely established for investigating these mechanisms in retinal pathology.

### 3.4 Oxidative stress mediates diabetes-induced retinal pigment epithelial cell damage

**Figure 4:**
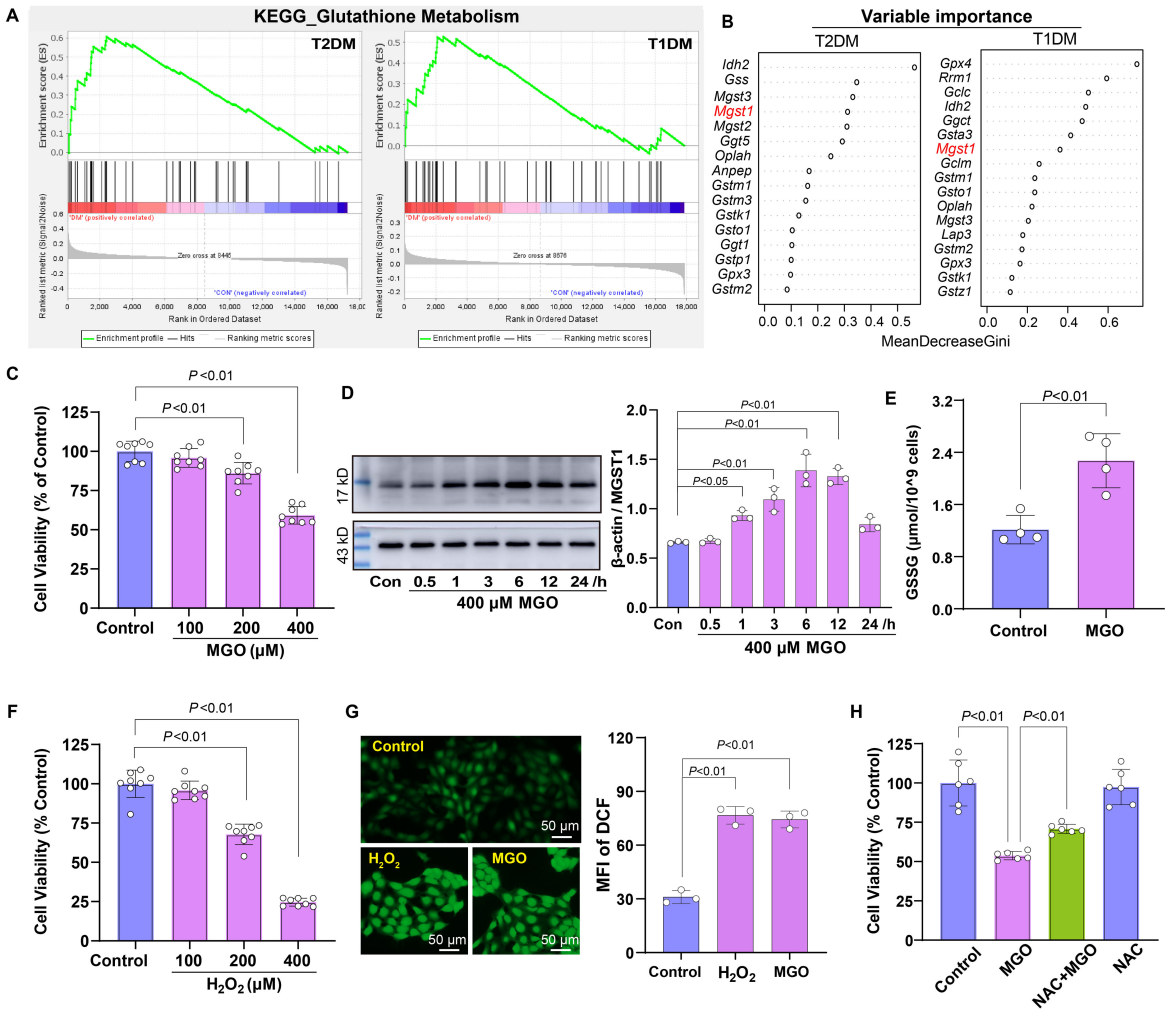
Roles of oxidative stress in diabetic retinopathy. **(A)** GSEA indicating enrichment of the Glutathione Metabolism pathway in retinal tissues of diabetic mice. **(B)** Random forest analysis identifying MGST1 as a key gene within this pathway. **(C)** Viability of ARPE-19 cells treated with MGO (100-400 μM) for 24 h was assessed using the CCK-8 assay. **(D)** MGST1 protein expression in ARPE-19 cells after MGO treatment was determined by Western blot. **(E)** Content of GSSG in ARPE-19 cells was measured after the treatment with 400 μM MGO for 24 h. **(F)** Viability of ARPE-19 cells after exposure to H_2_O_2_ (100-400 μM, 24 h) was evaluated as in **(C)**. **(G)** Intracellular ROS in ARPE-19 cells were visualized using DFCH-DH staining followed by fluorescence microscopy after exposure to 200 μM H_2_O_2_ or 400 μM MGO for 1 h. **(H)** ARPE-19 cells were treated with 400 μM MGO for 24 h with or without preconditioning with 2 mM NAC for 6 h, and viability was assessed as in **(C)**. Data are presented as mean ± SD, n = 3~8.

The enrichment analysis showed that the *Glutathione Metabolism* pathway was positively enriched in both types of diabetic retinal tissues (**Figure 4A**). To identify key regulatory genes within this pathway, random forest analysis was performed, which pinpointed MGST1 as a critical regulatory candidate, with IDH2 ranking higher than MGST1 (**Figure 4B**). MGST1, a mitochondrial glutathione S-transferase, plays a critical role in detoxifying ROS and carbonyl compounds, thereby protecting cells against oxidative and carbonyl stress. To investigate the role of oxidative stress in diabetic retinopathy, we established an *in vitro* model by exposing human retinal pigment epithelial ARPE-19 cells to MGO, a predominant precursor of AGEs in diabetic conditions. MGO treatment induced concentration-dependent cytotoxicity, with significant viability reduction at 200 and 400 μM (**Figure 4C**). Furthermore, MGO treatment induced a time-dependent upregulation of MGST1 protein expression over a 0–24 h exposure period (**Figure 4D**), suggesting that MGST1 may serve as an adaptive response to MGO-induced stress. Consistent with enhanced oxidative stress, MGO exposure resulted in significantly elevated GSSG levels, indicating GSH depletion and oxidative imbalance (**Figure 4E**). To validate the oxidative stress component, ARPE-19 cells were exposed to H_2_O_2_, which similarly reduced cell viability at 200 and 400 μM (**Figure 4F**). In addition, both MGO and H_2_O_2_ treatments markedly increased intracellular ROS content compared to control cells (**Figure 4G**). Notably, pretreatment with N-acetylcysteine, a GSH precursor, significantly attenuated MGO-induced damage (**Figure 4H**), demonstrating that MGST1 upregulation represents a compensatory antioxidant response to counteract oxidative injury in diabetic retinal cells.

### 3.5 p53-Mediated activation of DNA damage response in diabetic retina

**Figure 5:**
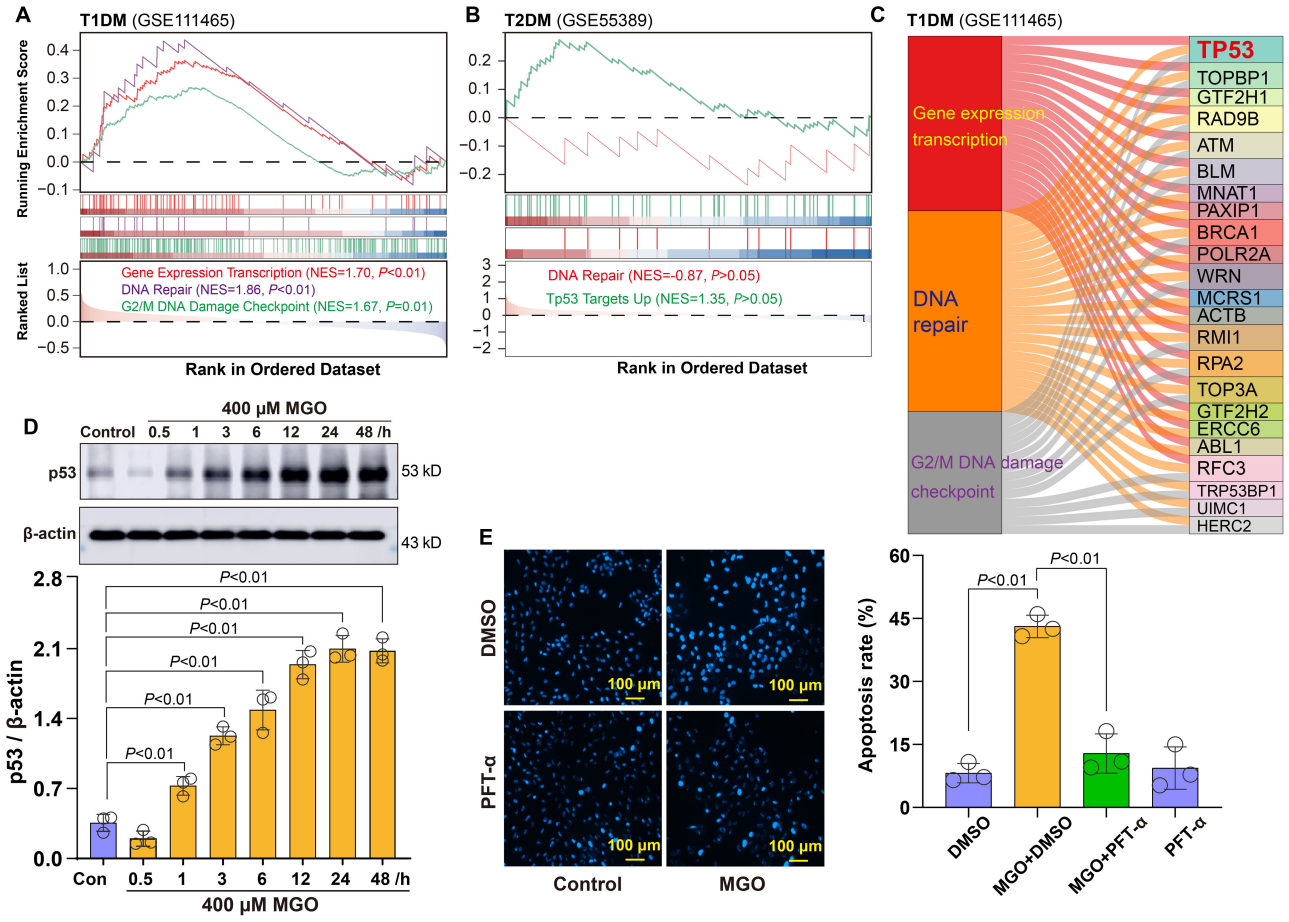
Roles of p53-related DNA damage in diabetic retinopathy. **(A)** GSEA showing significant enrichment of Gene Expression Transcription, DNA Repair, and G2/M DNA Damage Checkpoint pathways in retinal tissues from T1DM mice. **(B)** GSEA showing enrichment of DNA Repair and Tp53 Targets Up pathways in retinal tissues from T2DM mice. **(C)** Integrated network analysis revealing p53 as the central node. **(D)** Western blot analysis showing time-dependent upregulation of p53 expression in ARPE-19 cells treated with 400 μM MGO for indicated time points (1~48 h). **(E)** Apoptosis analysis of ARPE-19 cells treated with 400 μM MGO for 48 h in the presence or absence of the p53 inhibitor PFT-α at 20 μM.

Building upon the above observations of oxidative stress in diabetic retinal cells, we next investigated downstream cellular responses to such damage. GSEA revealed three significantly enriched pathways in T1DM retinal tissues: Gene Expression Transcription, DNA Repair, and G2/M DNA Damage Checkpoint (**Figure 5A**). In T2DM samples, Tp53 Targets and DNA repair pathways were also enriched, albeit to a lesser extent (**Figure 5B**). To identify key regulatory factors within these pathways, we performed integrated network analysis of genes implicated in these response mechanisms. This analysis identified Tp53 as the central node connecting these pathways (**Figure 5C**), suggesting its pivotal role in coordinating the cellular response to diabetic stress.

To validate this finding, we examined p53 protein expression in a cellular diabetic model. Western blot analysis demonstrated that the treatment of ARPE-19 cells with 400 µM MGO induced a time-dependent increase in p53 protein expression (**Figure 5D**), confirming activation of the DNA damage response under diabetic conditions. Furthermore, to assess the functional role of p53 in this context, we pretreated MGO-exposed ARPE-19 cells with the p53 inhibitor pifithrin-α (PFT-α). Notably, PFT-α treatment markedly suppressed MGO-induced apoptosis in retinal cells, as demonstrated by a significant reduction in the apoptotic rate compared to MGO alone group (**Figure 5E**). Collectively, these findings indicate that p53 functions not only as a hallmark of DNA damage responses but also serves as a critical pro-apoptotic regulator in diabetic retinal pathology.

### 3.6 Mitochondrial impairment in diabetic retinal tissues and IDH2-mediated adaptive protection

**Figure 6:**
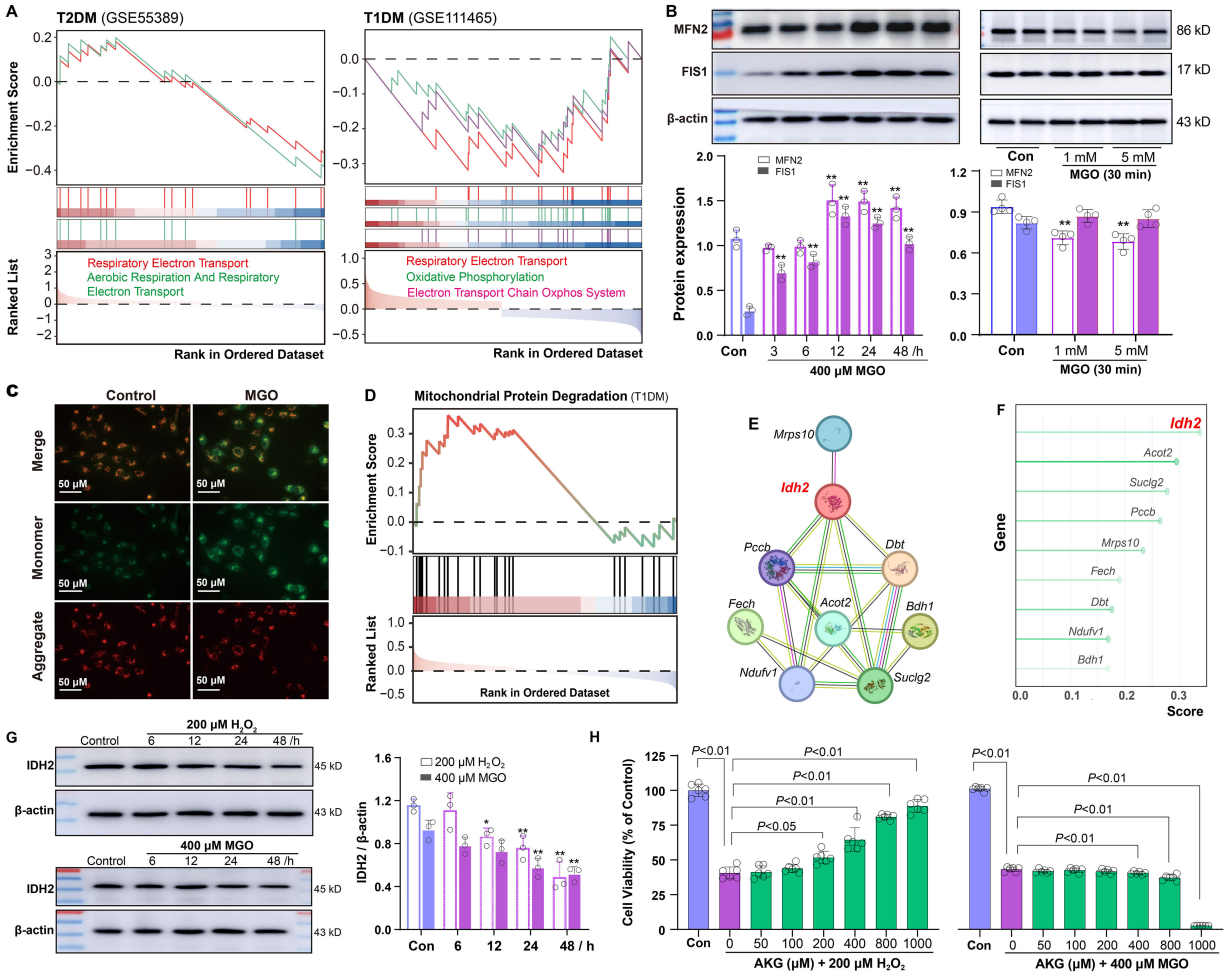
Mitochondrial impairment and IDH2 regulation in diabetic retinopathy. **(A)** GSEA showing negative enrichment of the mitochondrial electron transport chain in retinal tissues of T2DM and T1DM mice. **(B)** Western blot analysis of MFN2 and FIS1 in ARPE-19 cells following treatment with 400 μM MGO for 0~48 h or with 1~5 mM MGO for 30 min. **(C)** Mitochondrial membrane potential in ARPE-19 cells treated with 400 μM MGO for 24 h, was assessed using JC-1 staining and fluorescence imaging. **(D-F)** Enrichment analysis of genes involved in the mitochondrial protein degradation pathway in T1DM retinal tissues, network diagram illustrating protein-protein interactions among genes involved in this pathway, and random forest analysis ranking importance of the genes. **(G)** Western blot analysis of IDH2 in ARPE-19 cells treated with 200 μM H_2_O_2_ or 400 μM MGO for 0~48h. Quantification was performed by densitometric analysis and normalized to β-actin. **(H)** Viability of ARPE-19 cells treated with 200 μM H_2_O_2_ or 400 μM MGO with or without AKG (50~1000 μM), was assessed using the CCK-8 assay. Data are presented as mean ± SD, n = 3~6.

Given the association between oxidative stress and mitochondrial function, we examined mitochondrial integrity in diabetic retinal tissues. GSEA revealed significant impairment of the mitochondrial electron transport chain in both types of diabetic retinal tissues, with more pronounced dysfunction observed in T1DM (**Figure 6A**). To explore the temporal changes in mitochondrial dynamics under oxidative stress, ARPE-19 cells were treated with 400 µM MGO for 3, 6, 12, 24 and 48 h. Western blot analysis revealed a time-related increase in the expression of the mitochondrial fusion protein MFN2 and the fission protein FIS1 (**Figure 6B**), indicating an early compensatory response to stress. However, at higher MGO concentrations (1 and 5 mM), MFN2 expression was markedly reduced (**Figure 6B**), suggesting a shift toward mitochondrial fragmentation under severe stress conditions. Given the alterations in mitochondrial proteins, we assessed mitochondrial function by measuring membrane potential. It was found that MMP was significantly decreased in ARPE-19 cells following 400 µM MGO treatment (**Figure 6C**), as well as after 30 min treatment with high-concentration MGO (**Supplementary Figure S1**).

Building on these findings, we explored potential regulatory mechanisms. GSEA showed significant enrichment of *Mitochondrial Protein Degradation* pathway (**Figure 6D**). Additionally, protein-protein interaction analysis of genes associated with this phenotype identified IDH2 as a key bottleneck hub gene (**Figure 6E**). Further assessment using a random forest algorithm ranked IDH2 as the most critical gene (**Figure 6F**). To explore the role of IDH2 under different diabetic conditions, we analyzed its expression after ARPE-19 cells were exposed to H_2_O_2_ or MGO. IDH2 expression was significantly reduced in both conditions (**Figure 6G**). Moreover, cell viability assays demonstrated that supplementation with α-ketoglutarate (AKG), a catalytic product of IDH2, effectively mitigated H_2_O_2_-induced cell damage in ARPE-19 cells, but failed to rescue MGO-induced damage (**Figure 6H**).

### 3.7 Association of Keap1-Nrf2 pathway with sulfur-induced cytoprotection

**Figure 7:**
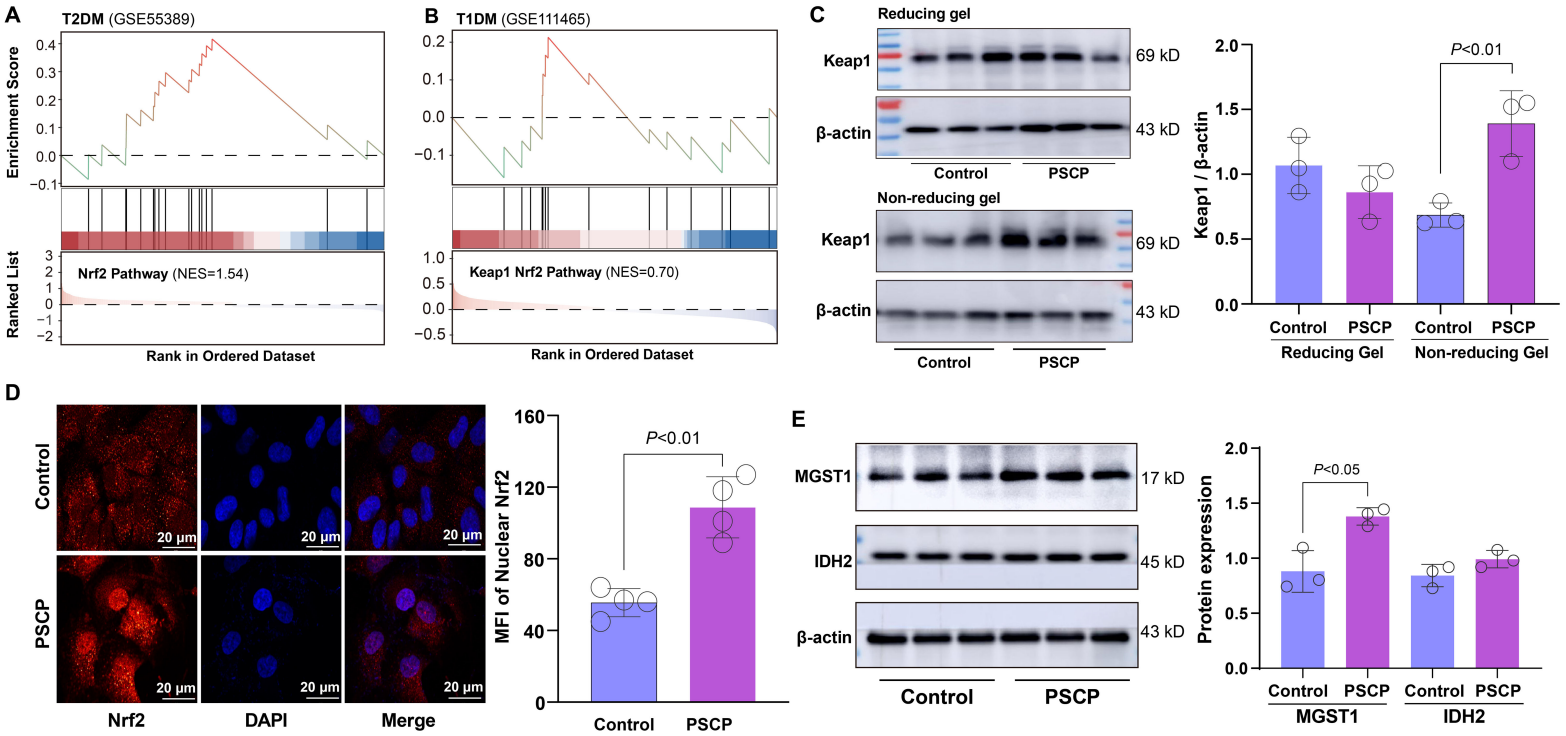
Effects of PSCP on keap1-Nrf2 pathway in retinal cells. **(A, B)** GSEA showing enrichment of Nrf2 pathway in retinal tissues from T2DM and T1DM mice. ARPE-19 cells were treated with 50 μM PSCP or vehicle control for 6 h, followed by: **(C)** Western blot analysis of Keap1 expression and tailing under reducing and non-reducing gel conditions; **(D)** Immunofluorescence combined with confocal microscopy to observe Nrf2 nuclear translocation; **(E)** Western blot analysis of MGST1 and IDH2 expression. Data are presented as mean ± SD (n=3~4).

GSEA revealed significant enrichment of the Nrf2 pathway in retinal tissues of T2DM mice with a NES of 1.54 (**Figure 7A**). In T1DM mouse retinal tissues, the Keap1-Nfe2l2 pathway (Nfe2l2 being the gene encoding Nrf2 protein) showed modest enrichment (**Figure 7B**). Given that reactive sulfur species are known activators of the Nrf2 pathway, we investigated whether exogenous sulfur supplementation could modulate this pathway in retinal cells. We treated ARPE-19 cells with 50 μM persulfided cysteine precursor (PSCP) for 6 h, a previously reported sulfur donor (16, 19), and observed pronounced Keap1 protein tailing under non-reducing gel conditions, while no changes were detected under reducing conditions (**Figure 7C**). This pattern indicated that Keap1 was post-translationally modified, likely through persulfidation of cysteine thiol groups. Such modifications interfered with the protein’s conformational stability or charge state, thereby affecting its migration pattern during electrophoresis and leading to the observed band tailing. Furthermore, the treatment with PSCP induced significant nuclear translocation of Nrf2, indicating activation of the Nrf2 pathway (**Figure 7D**), suggesting that the persulfidation of Keap1 may attenuate its inhibitory interaction with Nrf2 and facilitate its nuclear accumulation. Consistently, the expression of MGST1, a known Nrf2 target gene, was markedly upregulated following PSCP treatment, while IDH2 protein levels showed a modest increase (**Figure 7E**), further supporting the functional activation of the Nrf2 signal in retinal cells.

**Figure 8:**
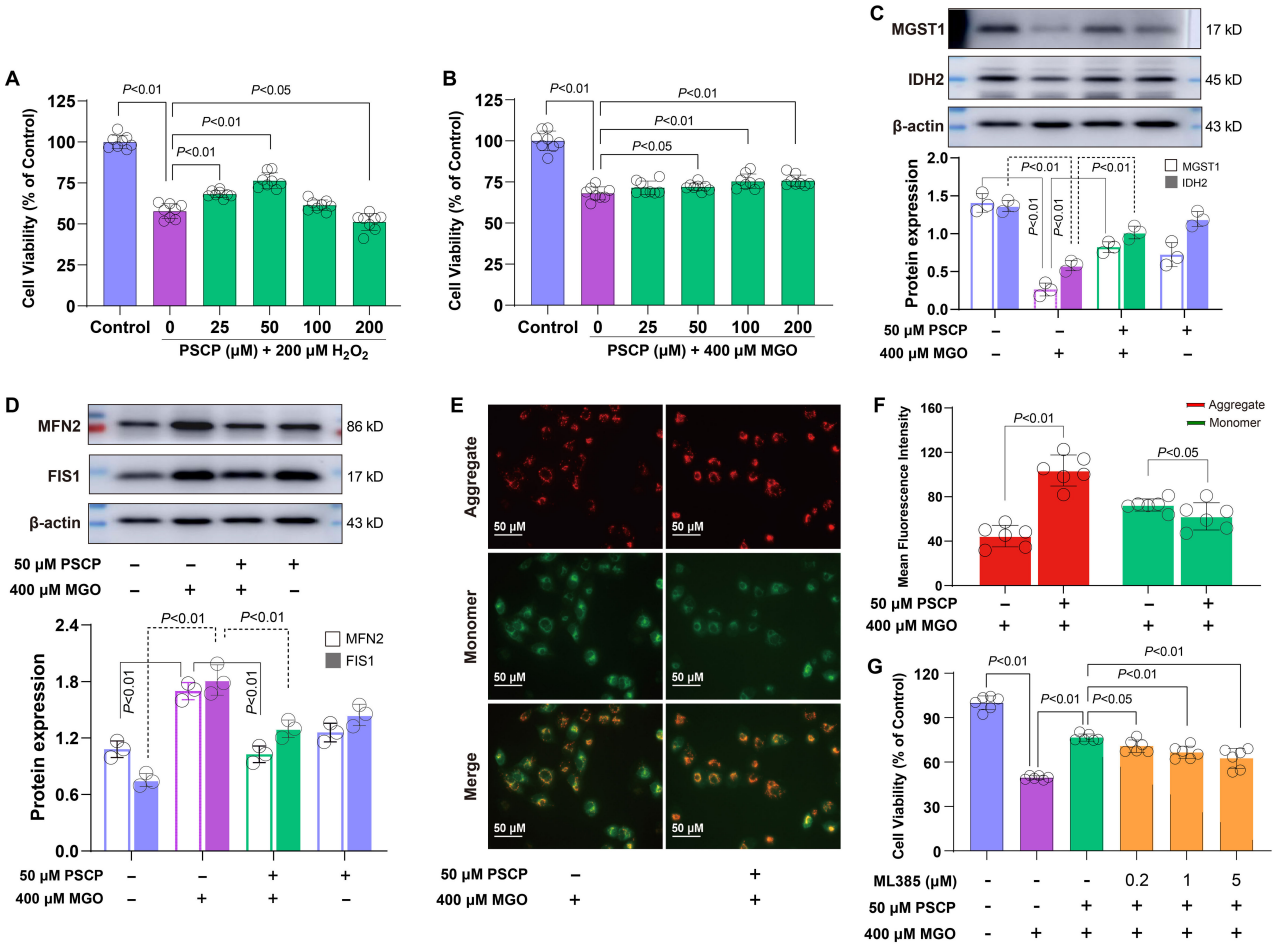
Roles of the keap1-Nrf2 pathway in PSCP-induced cytoprotection in retinal cells. **(A, B)** After the exposure of ARPE-19 cells to 200 μM H_2_O_2_ or 400 μM MGO for 24 h, with or without pretreatment with PSCP (25-200 μM) for 6 h, cell viability was determined with the CCK-8 assay. **(C)** Western blot analysis was used to measure the expression of MGST1 and IDH2 in ARPE-19 cells treated with 400 μM MGO for 24 h, in the absence or presence of pretreatment with 50 μM PSCP. **(D)** Following the same treatment protocol, the expression of MFN2 and FIS1 was assessed as in **(C)**. **(E, F)** After the treatment of ARPE-19 cells with 400 μM MGO for 24 h, with or without pretreatment with 50 μM PSCP for 6 h, mitochondrial membrane potential was evaluated using JC-1 staining and fluorescence imaging. **(G)** The ARPE-19 cells were exposed to 50 μM PSCP and 400 μM MGO for 24 h following pretreatment with ML385 (0.2-5 μM, 6 h), and cell viability was determined as in **(A)**. Data are presented as mean ± SD (n=3-6).

Based on these findings, we hypothesized that sulfur supplementation might attenuate diabetes-related retinal damage. To test this hypothesis, ARPE-19 cells were preconditioned with PSCP before exposure to either 200 μM H_2_O_2_ or 400 μM MGO. PSCP pretreatment showed a biphasic effect against H_2_O_2_-induced oxidative damage, with maximal protection observed at 25-50 μM, while 200 μM PSCP showed a slight reduction in cell viability (**Figure 8A**). Against MGO-induced carbonyl stress, PSCP provided modest but statistically significant protection (**Figure 8B**), suggesting differential efficacy depending on the type of metabolic stress. Mechanistically, PSCP inhibited MGO-induced downregulation of MGST1 and IDH2 (**Figure 8C**), and restored the aberrant upregulation of MFN2 and FIS1 (**Figure 8D**). More importantly, PSCP pretreatment improved mitochondrial membrane potential, suggesting a recovery of mitochondrial function (**Figures 8E, F**). Lastly, to confirm the role of Nrf2 in this protection, we employed the Nrf2 inhibitor ML385 (0.2-5 μM), which substantially reduced the protective effects of PSCP (**Figure 8G**). Collectively, these results demonstrate that activating the Keap1-Nrf2 pathway is critical for sulfur-induced protection in diabetic retinal tissues.

## 4 Discussion

In this study, we performed an integrative transcriptomic and cellular investigation to elucidate the distinct pathogenic mechanisms driving DR in type 1 and type 2 diabetes. Our results reveal that T1DM and T2DM induce divergent molecular and cellular stress responses in the retina, which contribute to their differing clinical presentations and progression patterns.

Transcriptomic clustering showed a stronger deviation of gene expression profiles in T1DM compared to T2DM, suggesting a more aggressive or acute transcriptional response to insulin deficiency. For example, T2DM retinas were enriched in neurodegenerative pathways, like AD and PD, indicating that retinal neuronal degeneration may be a key contributor to DR. In contrast, T1DM retinas showed significant enrichment related to DNA repair and p53 signaling. Notably, mitochondrial dysfunction, oxidative stress and Nrf2 signaling pathways were commonly enriched in both types of DM. This divergence suggests that T2DM-related DR may involve chronic neuronal compromise (6, 12, 20), and T1DM-associated DR engages a rapid and robust cellular stress response (21).

We speculate that the type-specific retinal damage in diabetes may arise from two mechanistic differences: First, T1DM primarily triggers metabolic stress through acute insulin deficiency and hyperglycemia, whereas T2DM involves chronic insulin resistance and systemic inflammation (2, 22). Second, neurodegeneration appears to be differentially regulated between diabetes types. T2DM retinas show upregulation of neurodegenerative pathways, possibly due to microglial activation, innate immune, and neuronal apoptosis, whereas these processes are not prominent in T1DM. This suggests that retinal neuronal degeneration in diabetic retinopathy may be driven by distinct mechanisms (23–25). These mechanistic differences may have important clinical implications: T1DM patients might benefit more from interventions targeting acute oxidative and genotoxic stress, while T2DM patients may require strategies that address chronic neuroinflammation and neurodegeneration. Understanding these type-specific pathogenic features could facilitate the development of personalized therapeutic approaches for diabetic retinopathy.

The consistent enrichment of GSH metabolism in both T1DM and T2DM retinas points to redox imbalance as a shared pathogenic hallmark of DR. Within this context, MGST1 emerges as a pivotal mitochondrial enzyme in outer membranes potentially linking carbonyl detoxification and redox homeostasis (26, 27). Its dynamic response to metabolic stress, particularly the biphasic regulation under MGO exposure, may reflect an adaptive but ultimately insufficient cellular defense mechanism (28). Given the observed protective effect of the application of GSH substrate (NAC), therapeutic strategies aimed at preserving or enhancing mitochondrial glutathione capacity, rather than simply quenching ROS, may offer more targeted protection against retinal injury in diabetes (11, 29).

In parallel, mitochondrial vulnerability represents another shared pathological axis in DR. IDH2, a mitochondrial NADP^+^-dependent isocitrate dehydrogenase localized to the mitochondrial matrix, plays a critical role in sustaining redox balance through NADPH regeneration (30). In our study, IDH2 expression was suppressed following both H_2_O_2_ and carbonyl MGO stress. However, only H_2_O_2_-induced injury was alleviated by α-ketoglutarate (AKG), the metabolic substrate of IDH2 (31–33). The failure of AKG to reverse MGO-induced damage suggests that carbonyl stress compromises mitochondrial function through mechanisms beyond redox depletion, such as irreversible protein carbonylation and AGE accumulation. These findings highlight a mechanistic divergence between oxidative and carbonyl stress. Specifically, while oxidative stress primarily induces reversible redox imbalance that can be mitigated by AKG, carbonyl stress causes irreversible post-translational modifications (e.g., AGE formation) that cannot be rescued by simply replenishing metabolic intermediates. This distinction underscores the need for differential therapeutic strategies: antioxidant or metabolic supplementation may suffice for oxidative injury, whereas carbonyl stress may require interventions that either prevent AGE formation or enhance protein quality control systems.

Transcriptomic analysis revealed significant enrichment of *Mitochondrial Protein Degradation* pathways and negative enrichment of electron transport chain-related genes, particularly in T1DM retinas, suggesting impaired mitochondrial quality control and energy metabolism. Functionally, we found that MGO treatment disrupted mitochondrial dynamics, as evidenced by altered expression of MFN2 and FIS1, and concomitantly suppressed MGST1 and IDH2, compounding mitochondrial vulnerability under carbonyl stress. MGST1 protects against lipid peroxidation injury in mitochondria (34), while IDH2 maintains mitochondrial redox integrity and supports respiratory chain activity (35). Our study highlights the concurrent suppression of these two enzymes in diabetic retinal stress, defining a mitochondrial collapse phenotype characterized by membrane depolarization—an integrative pathological feature not previously delineated in diabetic retinopathy.

Lastly, to investigate potential compensatory responses to mitochondrial dysfunction, we examined the Keap1-Nrf2 pathway, a critical antioxidant defense mechanism in DR (36), which showed transcriptional activation in both diabetic retinas. PSCP, a synthetic reactive sulfur compound we previously developed with demonstrated antitumor activity (16, 19), was applied to ARPE-19 cells. It induced Keap1 modification and promoted Nrf2 nuclear translocation, suggesting pathway activation. Similar modifications of Keap1 by other reactive sulfur species, including hydrogen persulfide and cysteine persulfide, have also been reported (37–39). It should be noted that while the observed electrophoretic mobility shift (“tailing”) of Keap1 suggests cysteine modification, direct biochemical identification of specific persulfidation sites by mass spectrometry was not performed in this study. Further validation using modification-specific antibodies or proteomic approaches would strengthen the mechanistic understanding of PSCP-Keap1 interactions. Importantly, PSCP treatment restored the expression of MGST1 and IDH2, which was suppressed under MGO-induced stress, accompanied by improved cell viability and normalization of mitochondrial dynamics. These protective effects were abolished by ML385, the Nrf2 inhibitor (40), confirming that Nrf2 plays an important role in mediating the response. Although direct regulation of MGST1 and IDH2 by Nrf2 was not observed in this study, both genes are well-established Nrf2 targets (41, 42), which reinforces the proposed mechanistic link between sulfur signaling and mitochondrial resilience.

Several limitations should be acknowledged. While our findings provide mechanistic insights into PSCP-mediated protection, the current study was designed as a hypothesis-generating investigation based on transcriptomic analysis and *in vitro* experiments, and *in vivo* validation was beyond its scope. Additionally, validation of IDH2 and MGST1 expression in human diabetic retinal tissues was not performed due to the significant challenges in obtaining such samples. Moreover, the exclusive use of ARPE-19 cells, while appropriate for modeling retinal oxidative stress, may not fully recapitulate the complexity of retinal microenvironment. The pharmacological properties of PSCP, including its stability, bioavailability, and cell-type specificity in retinal tissue, also remain to be systematically characterized. Future studies using diabetic animal models with functional and structural assessments, diverse retinal cell types (e.g., retinal ganglion cells and endothelial cells), comprehensive pharmacokinetic and pharmacodynamic profiling of PSCP, as well as clinical sample validation, are warranted to confirm the translational potential of these findings.

## 5 Conclusions

Our findings reveal a dual-stress pattern in diabetic retinopathy: mitochondrial compromise marked by IDH2 and MGST1 suppression, and a sulfur-driven antioxidant defense through Nrf2 activation. This redox-mitochondrial axis offers new insight into disease heterogeneity and suggests PSCP as a potential therapeutic strategy targeting mitochondrial vulnerability in diabetes. However, further investigations into the pharmacokinetic properties, potential toxicity, and retinal tissue specificity of PSCP are necessary before clinical translation. Our study provides a mechanistic foundation for future preclinical and clinical development of sulfur-based therapeutics in diabetic retinopathy.

## Data Availability

Publicly available datasets from the GEO database were analyzed in this study. No new datasets were generated.
