# Supplementary figures and images for: PSCP, a novel reactive sulfur donor, activates Keap1-Nrf2 signaling and attenuates mitochondrial dysfunction in diabetic retinopathy
Source: Front Endocrinol (Lausanne). 2025 Nov 17;16:1690553. doi: 10.3389/fendo.2025.1690553 (PMC12665532; doi:10.3389/fendo.2025.1690553)

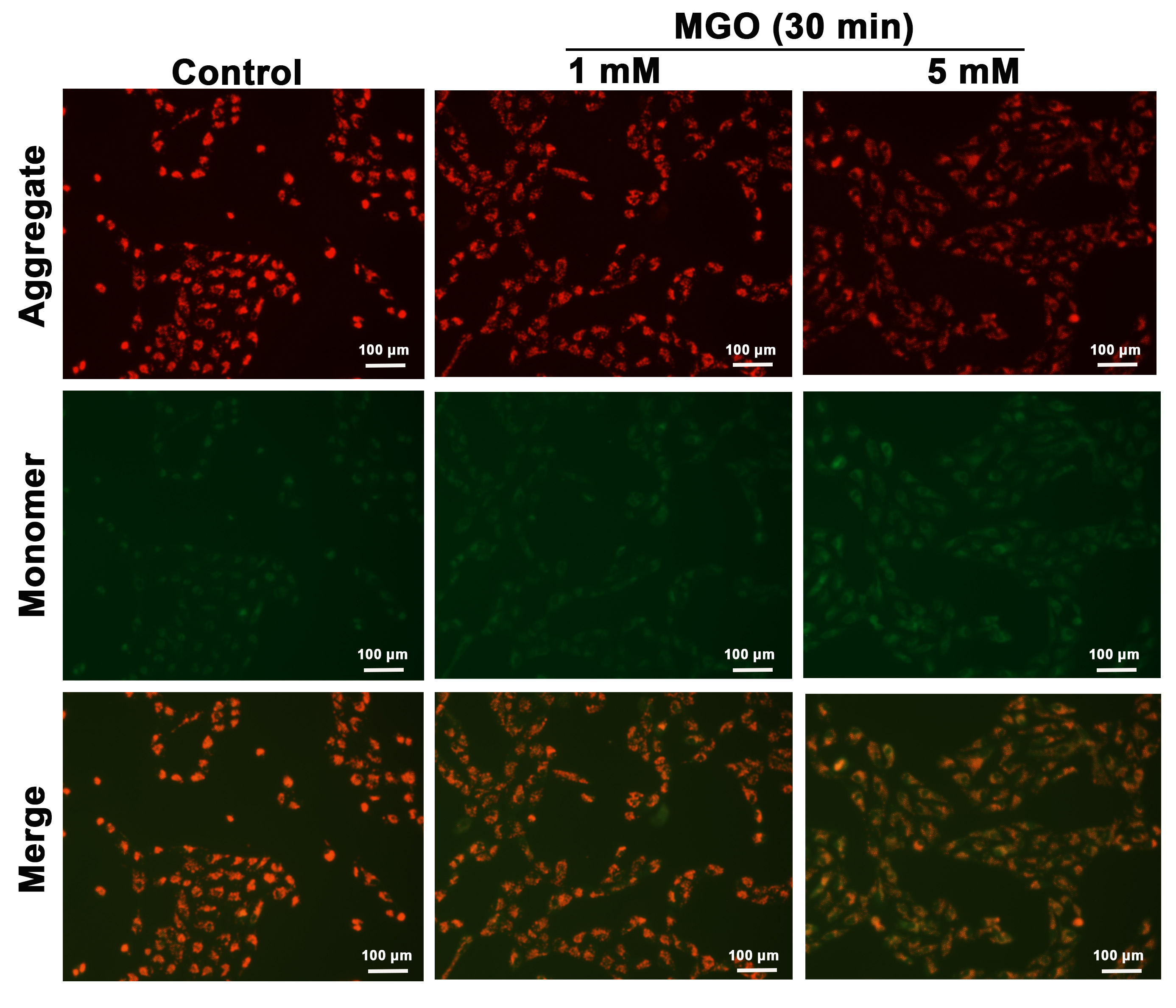

Supplement: Supplementary file 1 [file Image1.jpeg]
